# Housing Conditions, Neighborhood Physical Environment, and Secondhand Smoke Exposure at Home: Evidence from Chinese Rural-to-Urban Migrant Workers

**DOI:** 10.3390/ijerph17082629

**Published:** 2020-04-11

**Authors:** Chenghan Xiao, Yang Yang, Xiaohe Xu, Xiao Ma

**Affiliations:** 1 West China School of Public Health, Sichuan University, Chengdu 610041, China; chenghan_xiao@hotmail.com (C.X.); yangyang@scu.edu.cn (Y.Y.); 2 School of Public Administration, Sichuan University, Chengdu 610065, China; xiaohe.xu@utsa.edu; 3 Department of Sociology, University of Texas at San Antonio, TX 78249, USA

**Keywords:** secondhand smoke, rural-to-urban migrant workers, housing conditions, neighborhood physical environment

## Abstract

Over the past two decades, health-related issues among rural-to-urban migrant workers in China have been widely discussed and documented by public health scholars. However, little, if any, scholarly attention has been paid to migrant workers’ secondhand smoke (SHS) exposure at home. This study aims to explore the contours of SHS exposure at home and investigate the effects of inadequate housing conditions and poor neighborhood physical environments on such in-home exposure among Chinese migrant workers. A respondent-driven sampling method was employed to interview 1854 rural-to-urban migrant workers from the period June 2017 to June 2018 in Chengdu, China. The results indicate that Chinese migrant workers are at high risk of SHS exposure at home. Migrant workers who live in homes with inadequate conditions, such as substandard housing and crowdedness, are especially at high risk of SHS exposure at home. Moreover, poor neighborhood physical environments are significantly and positively associated with SHS exposure at home. These findings suggest that strategies that can help improve housing conditions and neighborhood physical environments should be developed and promoted to protect rural-to-urban migrant workers from SHS exposure at home.

## 1. Introduction

No risk-free level of secondhand smoke (SHS) exposure exists [1]. Even brief exposure can depress the endothelial function, thereby giving rise to long-term vascular damage [2,3]. Past research has documented that the SHS exposure is associated with a wide array of negative health outcomes, such as lung cancer, respiratory symptoms, ear infections, and asthma attacks [4,5]. Given these shreds of evidence, many countries have enacted laws prohibiting smoking in public places to control and reduce SHS exposure. Even though bans on smoking in public places have significantly reduced overall exposure, studies indicate that the prevalence of SHS exposure in homes remains high [6]. This is particularly true for people who live in poverty and/or reside in rental housing [7]. With regard to China, the extant literature on smoke-free homes suggests that setting smoke-free rules and persuading smoking cessation are perhaps the most effective ways to control SHS exposure at home [8]. However, studies caution that almost no Chinese families with low-income and insufficient-education can conduct complete smoking bans in their homes [9,10]. Others also argue that implementing smoking ban rules at home can be socially challenging and costly [11,12].

With these divergent views in mind, the present study is designed to explore the contours of SHS exposure at home in a specific subpopulation in urban China, namely, rural-to-urban migrant workers. Given their low socioeconomic status and dependency on rental housing, coupled with prevalent tobacco use [13,14,15], Chinese rural-to-urban migrant workers are at a greater risk for SHS exposure at home. Surprisingly however, little scholarly attention has been given to such an important public health problem in this subpopulation despite the fact that there are more than 288 million migrant workers who live and work in today’s urban China [16]. To move beyond a limited body of research that explored associations between SHS exposure and demographic factors among migrant workers in China [17,18], this study focuses sharply on how (1) inadequate housing conditions and (2) neighborhood environmental problems associated with SHS exposure at home among Chinese rural-to-urban migrant workers.

### 1.1. Poor Housing Conditions and Secondhand Smoke Exposure at Home

Prior studies have documented that low per capita living space, crowded housing, inadequate facilities, and poor neighborhood physical environments are the major disadvantages confronted by Chinese migrant workers [15,19,20]. Due to their disadvantaged socioeconomic positions, migrant workers are often forced to choose crowded dormitories or rental housing without sufficient facilities as their main residence [21]. It is not uncommon for migrant workers to live in “urban villages” located in the fringe areas of cities, which are thought of as ghettos associated with unplanned land use, decaying housing conditions, reduced public safety, deteriorating social order, and little vegetation [19,22]. Such residential inequalities endured by Chinese migrant workers are similar to the residential segregation experienced by African Americans in the twentieth century in America [23,24].

The relationship between inadequate housing conditions and SHS exposure at home has been investigated in various subpopulations. Studies conducted in the United States reported that people living in multiunit housing with a smoke-free policy are still vulnerable to SHS exposure due to the inability to prevent smoke transfer [25,26]. Crowdedness and houses that do not have sufficient rooms are also reportedly associated with higher levels of SHS exposure at home for children [27]. Beyond the physical incursion or transpiration of smoke, extant studies have also noted psychological factors. For example, stress has been identified as a contributing factor to SHS exposure at home in relation to poor housing conditions [28], as smoking has been frequently used as one of the stress-coping mechanisms [29,30]. Consequently, people living in such homes might be disproportionately exposed to higher levels of SHS.

### 1.2. Poor Neighborhood Physical Environments and Secondhand Smoke Exposure at Home

The association between poor neighborhood physical environments and SHS exposure at home has been largely overlooked by literature on smoke-free homes. Only a handful of studies conducted recently have begun to investigate and report this unique association [31]. Researchers have argued that the association between poor neighborhood physical environments and SHS exposure at home could be due to smoking behavior, a pivotal factor in SHS exposure at home [32,33]. Reminiscent of inadequate housing conditions, poor neighborhood physical environments can lead to higher levels of stress perception, thus contributing to smoking in-home and unsuccessful attempts to quit smoking [34,35]. Some scholars have also found that neighborhoods with a high density of vegetation can alter smoking behavior and reduce smoking by developing and building stronger social cohesion [36,37]. In contrast, in neighborhoods with a high rate of crime, residents may smoke more indoors since the crime and environmental concerns can make residents feel unsafe and unpleasant to smoke outside [33]. With these burgeoning study findings, it is plausible to hypothesize an association between neighborhood physical environments and SHS exposure at home due largely to the impact of a poor neighborhood physical environment on smoking behavior.

To summarize, the SHS exposure at home among Chinese rural-to-urban migrant workers is unclear, and the extent to which inadequate housing conditions and neighborhood environmental problems are associated with SHS exposure at home is understudied. It is in this context that the present study can make several unique contributions to literature on SHS exposure at home. First, this study explores the contours of SHS exposure at home among non-smoking rural-to-urban migrant workers in contemporary China. Second, this study examines the association between inadequate housing conditions, neighborhood environmental problems, and SHS exposure at home among non-smoking rural-to-urban migrant workers. Finally, this study utilizes the spatial scan and sensor data analytical tools to supplement conventional multivariate regression analysis. 

## 2. Materials and Methods 

### 2.1. Sample and Data Collection

Data for this study came from a cross-section survey that collected a wide range of health-related information from migrant workers in Chengdu, China—the capital of Sichuan province. The respondent-driven sampling (RDS) technique was employed to recruit qualified migrant workers from the period June 2017 to June 2018. RDS is a sampling method that features a subject-referred or chain referral procedure, which is a popular sampling technique utilized by researchers to collect information from various hard-to-reach populations [38]. The chief merit of this chain sampling procedure is that it allows researchers to provide unbiased population estimates and measure the precision of those estimates through analytical tools [39,40].

Specifically, 12 migrant workers were first selected to serve as the “seeds” of the 12 potential chain waves reflecting such characteristics as gender, age, occupation, and place of residence. After face-to-face interviews, all “seeds” were trained and encouraged to recruit another three migrant workers from their respective social networks. The newly recruited migrant workers from these “seeds” were then interviewed, trained, and asked to recruit three more qualified migrant workers. A dual incentive system was utilized to stimulate participation and recruitment. First, all survey participants received a “participation incentive” of RMB50 (about US$7.1) as compensation for their time spent in the interview. Second, all participants also received a “recruitment incentive” of RMB10 (about US$1.4) if they successfully recruited one qualified migrant worker from their social networks. The maximum of the “recruitment incentive” was RMB30 (about US$4.2), as every participant could recruit up to three qualified migrant workers. This sampling process was repeated until the sample reached the point of equilibrium. It is important to note that this procedure yielded an unbiased sample of migrant workers, even though the sampling process started with an arbitrarily chosen set of initial seeds [38]. Interviews were conducted at the migrant workers’ homes, workplaces, or in the research offices across the city. Informed consent was obtained from all participants before they were included in the study. The Medical Ethics Committee at Sichuan University approved this survey project (K2017033-02).

Unexpectedly, however, three of the 12 chains recruited fewer than 15 qualified migrant workers because the first or second wave of respondents in these three chains declined to recruit more participants from their social networks. As a result, nine remaining chains were deemed valid for the present study. The estimated discrepancies between the actual and equilibrium sample compositions were 0.20%, 1.81%, and 1.50%, respectively, for gender, age, and occupation. These values are smaller than the suggested threshold of 2%, indicating that the collected sample reached equilibrium. The present study included a total of 1854 rural-to-urban migrant workers with complete survey information. Out of these migrant workers, 1251 non-smoking respondents were identified and utilized as a subsample to examine the association between inadequate housing conditions, neighborhood environmental problems, and SHS exposure at home.

### 2.2. Variable

SHS exposure at home, serving as the dependent variable for this study, was measured by a global survey question, which asked the respondents, “In general, how many days you are exposed to secondhand smoke at home or inside the building every week?”. Responses to this question were recoded into four rank-ordered categories with 0 = never, 1 = 1–3 days per week, 2 = 4–6 days per week, and 3 = every day.

To gauge housing conditions as a focal independent variable, respondents were asked to report the following: (1) housing type, (2) residential crowdedness, and (3) availability of essential facilities. Housing type was dummy-coded into 0 = storied commercial building and 1 = unusual type (including shanties, prefabricated houses, or self-constructed bungalows). The crowdedness was also dummy-coded with 0 = two or fewer people sharing one bedroom and 1 = three or more people sharing one bedroom. The availability of essential facilities was measured by respondent’s report on the availability of a kitchen, a living room, and/or a restroom. The absence of at least two of the three above facilities was coded as 1 = lack of essential facilities and 0 = availability of essential facilities.

Another focal independent variable is the neighborhood physical environment. Respondents were first asked about their perceptions about their neighborhood hygiene and noise problems. Hygiene problems included the presence of rats, uncollected litter, and polluted water in the neighborhood. Noise problems were simply estimated by the respondent’s perception of the noise level in the neighborhood. All responses were recorded on five-point Likert scales, with higher values indicating more severe neighborhood environmental problems. Moreover, a spatial scan statistic based on the self-reported problems as mentioned above was estimated to identify significant cluster areas within which neighborhood environmental problems exist. Thus, respondents living inside the identified cluster areas were coded as living in the neighborhoods that had hygiene and/or noise problems. 

In addition to the spatial scan statistic, the sensor tool was used to measure the objective neighborhood greenness, which is a crucial component of the neighborhood physical environment. The Normalized Difference Vegetation Index (NDVI) values from the Moderate-Resolution Imaging Spectroradiometer (MODIS) were estimated to measure the objective neighborhood greenness [41]. The NDVI value ranges from −1 to +1, with higher values indicating more green space [42]. MODIS is an instrument aboard the Terra satellite which can offer global 250 m × 250 m pixel NDVI value every 16 days [43]. This study created a set of 800 m radius buffer zones according to the residential addressees of respondents to present the neighborhood range of every respondent [44,45]. The neighborhood greenness was measured according to the mean 2018 annual average NDVI value for all 250 m × 250 m pixels within their 800 m buffers. Finally, respondents were dummy-coded as 0 if there was an NDVI value less than 0.3 and 1 if there was an NDVI value more than 0.3, since the low and high density of vegetation can generally be divided by 0.3 [46]. The above geographic information system analyses were conducted through ArcMap 10.2. 

This study also controlled for a series of potential confounding factors. These factors included gender (0 = male, 1 = female), age (in years), annual income from all sources in RMB (log-transformed), marital status (0 = married, 1 = not in a marital union), education level (from 0 = no formal education to 5 = college and above), occupation (0 = construction industry, 1 = manufacturing industry, 2 = service industry, and 3 = others), living with children (0 = no, 1 = yes), and the harmfulness of SHS perceived by the respondents (0 = no, 1 = yes).

### 2.3. Statistical Analyses

A spatial scan statistic for continuous data was used to detect whether there were geographical cluster areas with the reported hygiene and/or noise problems, respectively, in the residential neighborhoods where the respondents resided. The input data were self-perceived neighborhood hygiene and noise problems reported by all the 1854 respondents, with spatial latitude and longitude drawn from their home addresses. A set of circles with radii varying from 0 km up to 2 km were built to detect the cluster areas. The test statistic was defined as the maximum log-likelihood ratio over every circle. The specific procedure of this spatial scan statistic proposed by Martin et al. (2009) was followed [47]. Fishnet maps were applied to display the geographical cluster results by dividing the square of five urban districts and five suburban counties of Chengdu city in 100 × 100 fishnets. Each sample or cluster was located in a fishnet according to its latitude and longitude. “No data area” meant that there was no sample available in that location. SaTScan 9.4 (Martin Kulldorff, Boston, USA) was used to implement the spatial scan data processing, while ArcMap 10.2 (Esri China Information Technology Co. Ltd., Beijing, China) was utilized to make the fishnet map. 

Ordered logistic regression models were estimated to establish regression relationships between inadequate housing conditions, neighborhood environmental problems, and SHS exposure at home among Chinese rural-to-urban migrant workers. Ordered logistic regression models without confounding factors were first estimated to generate the unadjusted odds ratio (OR) for every single poor housing condition and neighborhood environmental problem. The partially adjusted OR for every single poor housing condition and neighborhood environmental problem was then estimated through ordered logistic regression models net of the confounding factors mentioned above. Finally, two fully adjusted ordered logistic regression models were estimated with one including all the independent and control variables with the exception of the spatial scan statistic and the other containing all the independent and control variables but excluding the self-perceived neighborhood environmental problem variables. All ordered logistic regression models were estimated using the package of “MASS” in R 3.6.1 (R Foundation for Statistical Computing, Vienna, Austria).

## 3. Results

### 3.1. Secondhand Smoke Exposure

Table 1 displays the descriptive statistics of SHS exposure at home and sociodemographic characteristics for all respondents and non-smoking respondents separately. Of the 1251 rural-to-urban migrant workers who are non-smokers, about 45% reported that they were aware of SHS at home on some days or every day. This figure is lower than rural residents but higher than urban residents in China [48,49]. Moreover, the majority of migrant workers perceived SHS exposure as harmful. Specifically, about 86% of all rural-to-urban migrant workers and 87% of non-smoking migrant workers viewed SHS exposure as harmful to health. 

### 3.2. Housing Conditions and Neighborhood Physical Environments

As shown in Table 1, about 63% of all migrant workers resided in storied commercial housing units, while around 37% lived in unusual housing types, such as shanties, prefabricated houses, and self-constructed bungalows. Furthermore, about 29% of all migrant workers reported sharing a bedroom with at least three roommates. Nearly 44% of all rural-to-urban migrant workers resided in housing units that did not have adequate facilities, such as a kitchen, a living room, and/or a restroom. By contrast, the housing conditions for non-smoking rural-to-urban migrant workers were slightly better than all migrant workers combined. However, they too faced similar poor housing conditions such as residing in unusual types of housing units and crowded bedrooms with the absence of basic indoor facilities.

Table 1 also features the descriptive results of perceived neighborhood environmental problems. On a scale from one to five, the average levels of having neighborhood hygiene problems such as the presence of rats, uncollected litter and polluted water were 2.40 and 2.41 for all respondents and non-smoking respondents (“somewhat often” for both groups), respectively. However, noise seemed to be a common problem. About 60% of all respondents and non-smoking respondents (not shown in the table) reported experiencing excessive noise in their neighborhoods with the average levels of noise being 2.88 and 2.86 for all respondents and non-smoking respondents, respectively, on a scale from one to five (“often” for both groups).

**Figure 1:**
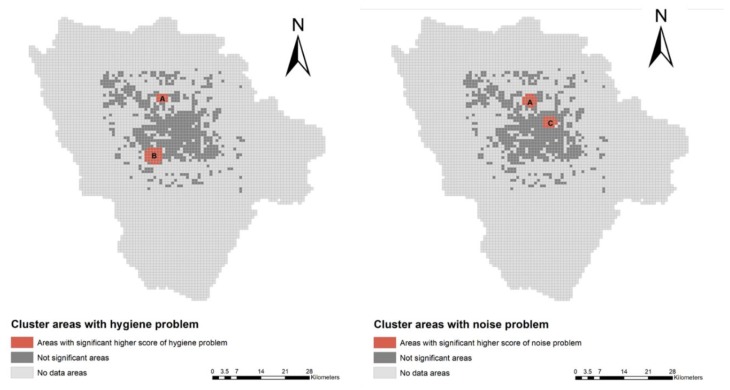
Spatial scan statistic results displaying cluster areas with hygiene and/or noise problems.

Figure 1 shows the significant cluster areas with both hygiene and noise problems identified by the scan statistics. As suggested by the figure, migrant workers living in area A reported experiencing both poor hygiene and high-level noise problems simultaneously in their neighborhoods, whereas migrant workers in area B and area C reported experiencing either a hygiene problem or a noise problem. Consistent with our fieldwork, area A and area B are identified as typical urban villages in the city of Chengdu, whereas area C contains numerous construction sites.

**Figure 2:**
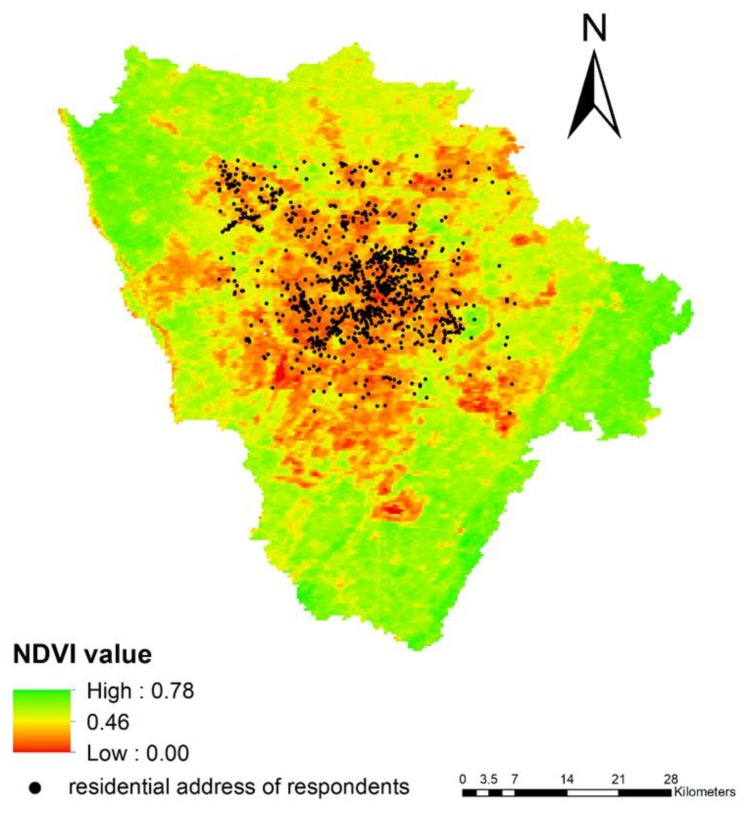
The NDVI value and secondhand smoke (SHS) exposure at home among non-smoking migrant workers. NDVI: Normalized Difference Vegetation Index.

Figure 2 illustrates the mean NDVI value of Chengdu in each 250 m × 250 m pixel. The mean NDVI value of the 800 m radius buffers ranged from 0.219 to 0.645, and the median and mean values were 0.308 and 0.321 (not shown in the figure), respectively. This visual display is highly consistent with the descriptive results reported in Table 1, which indicates that about 45% of all migrant workers and 44% of non-smoking migrant workers lived in an area with sparse vegetation or greenness (with a mean NDVI value of less than 0.3).

### 3.3. Results from Logistic Regression Models

Table 2 reports the unadjusted ORs of housing conditions and neighborhood physical environments while predicting SHS exposure at home among non-smoking migrant workers. The results suggest that all inadequate housing conditions significantly increase the odds of higher SHS exposure at home, and all favorable neighborhood physical environments significantly decrease the odds of higher SHS exposure at home among non-smoking migrant workers.

After controlling for potential confounding factors such as gender, age, marital status, education, occupation, income, living with children, and perceived harmfulness of SHS exposure, the association between each of the inadequate housing conditions, favorable neighborhood physical environments, and SHS exposure at home among non-smoking migrant workers remains statistically significant (see Table 3). The lone exception is that the neighborhood vegetation variable is no longer significantly associated with SHS exposure at home.

Table 4 reports the regression results by excluding the spatial scan statistics. Results indicate that unusual housing type (OR: 1.68, 95% CI: 1.22-2.31), sharing a bedroom with more than three people (OR: 1.73, 95% CI: 1.26-2.38), perceived neighborhood hygiene problems (OR: 1.17, 95% CI: 1.02-1.34), and perceived neighborhood noise problems (OR: 1.13, 95% CI: 1.01-1.27) are all significantly associated with the odds of higher SHS exposure at home among non-smoking migrant workers.

Table 5 displays the regression results by excluding the perceived neighborhood environmental problem variables. The association between housing conditions and SHS exposure at home is similar to those reported in Table 4. Unusual housing type (OR: 1.64, 95% CI: 1.19-2.27) and sharing a bedroom with more than three people (OR: 1.72, 95% CI: 1.30-2.38) are still significantly and positively associated with the odds of higher SHS exposure at home among non-smoking migrant workers. In addition, non-smoking migrant workers living outside of the cluster areas with poor hygiene problems are less likely to be exposed to SHS at home (OR: 0.62, 95% CI: 0.41-0.95). Likewise, non-smoking migrant workers living outside of the cluster areas with excessive noise are less likely to be exposed to SHS at home as well (OR: 0.58, 95% CI: 0.36-0.93).

## 4. Discussion

This study sheds new light on the effects of inadequate housing conditions and poor neighborhood physical environments on SHS exposure at home among non-smoking migrant workers in Chengdu, China. In the pages that follow, three noteworthy findings are reiterated and further discussed. Study limitation and directions for future research are offered. In light of these study findings and limitations, policy implications and recommendations are briefly discussed.

First, our findings revealed that SHS exposure at home among Chinese rural-to-urban migrant workers was prevalent. About 45% of non-smoking migrant workers included in this study reported being frequently exposed to SHS at home, ranging from one to three days per week to daily exposure. Even though this prevalence level is lower than the estimated value of 59% for the rural population [49], it is nevertheless higher than the estimated prevalence of 35% for the urban population [48]. Not only does this finding provide another important indicator of health disparity between the rural and urban populations in China, but it also characterizes the transitional nature of the subpopulation of rural-to-urban migrant workers in terms of serious public health concerns. 

Second, our results are partially consistent with previous research findings pertaining to inadequate housing conditions, such as per capita living space, density and crowdedness and essential facilities, and neighborhood environmental problems [15,20]. More than a quarter of migrant workers in this study reported sharing their bedroom with more than two individuals, and about 43% indicated that they lived in a housing unit without essential facilities, such as a kitchen, a living room, and/or a restroom. Moreover, more than one-third of migrant workers reportedly lived in shanties, prefabricated houses, or self-constructed bungalows. Additionally, it was common for migrant workers to reside in disadvantaged neighborhoods or urban villages that were marked by serious hygiene problems and/or excessive noise. In other words, geospatially migrant workers in this study tended to reside in cluster areas that were characterized by different types of neighborhood environmental problems. Finally, sensor data suggested that a good portion of migrant workers were likely to live in neighborhoods that had a low density of vegetation or greenness.

Third, our findings derived from a series of ordered logistic regression models demonstrated that the self-reported poor housing conditions and poor neighborhood physical environments significantly contributed to SHS exposure in homes among non-smoking migrant workers. While the significant association between inadequate housing conditions and SHS exposure at home lent credence to previous studies [26,27], a novel finding from this study was that perceived neighborhood environmental problems were also significantly associated with SHS exposure at home among non-smoking migrant workers. These results confirmed limited study findings that suggested a link between neighborhood environmental problems and SHS exposure at home [31,33]. More interestingly, using the spatial scan statistics to detect cluster areas marked by either hygiene or noise problems or both, migrant workers who resided outside the cluster areas were less likely to be exposed to SHS at home. However, it must be noted that after controlling for sociodemographic characteristics, the association between neighborhood greenness and SHS exposure at home was no longer statistically significant. 

The findings reiterated above suggest that allocating resources to improve housing conditions and neighborhood physical environments for migrant workers might be a supplementary strategy to address the SHS exposure at home. While implementing smoking ban rules in homes is a necessary step toward reducing indoor SHS exposure, to successfully adopt these rules and regulations for migrant workers can be difficult and costly [11,12]. However, if the alternative strategy suggested here is proven to be practical and feasible, it should be implemented simultaneously along with other policies and programs that are already in place.

Several study limitations and directions for future research should be acknowledged. First of all, the measure of SHS exposure at home included in this study was self-reported, which might be subject to recall bias. To address this potential bias, objective air-monitoring measures or devices are recommended for future research in conjunction with self-reported measures. Secondly, resembling many observational studies, this research utilized a cross-sectional survey. As such, the reported associations between inadequate housing conditions, neighborhood environmental problems, and SHS exposure at home do not imply causal relationships. To critically examine and establish such causations, future studies are encouraged to collect longitudinal data with appropriate temporal order and measures. Third, the survey used in this study did not collect information regarding the number of smokers in the home and access to permitted smoking locations or places. Consequently, these potential confounders could not be controlled for in the multivariate regression analysis. Last but not least, the results reported in the present study may not be generalizable to geographical locations other than southwest China or Sichuan province due to the heterogeneity of Chinese provinces and cities. Therefore, replication studies in other regions of China are encouraged in future research.

## 5. Conclusions

Chinese rural-to-urban migrant workers are at a high risk for SHS exposure in their homes. As anticipated, risk factors such as inadequate housing conditions and poor neighborhood physical environments significantly contribute to non-smoking migrant workers’ SHS exposure at home. In addition to urging migrant workers to adopt smoking ban rules in their homes, housing reform and neighborhood environment amelioration might be fruitful as supplementary measures to help address the SHS exposure at home among rural-to-urban migrant workers in contemporary China.

## Figures and Tables

**Table 1:** Sociodemographic characteristics of respondents.

Variables	All Respondents		Non-Smoking Respondents	
	n	Percentage	n	Percentage
Gender				
Male	948	51.13	418	33.41
Female	906	48.87	833	66.59
Age ^1^	1854	41.92 (13.09)	1251	41.68 (13.09)
Marital status				
Married	1438	77.56	1003	80.18
Not in a marital union	416	22.44	248	19.82
Education ^1^	1854	3.02 (1.11)	1251	3.02 (1.13)
Occupation				
Construction industry	346	18.66	152	12.15
Manufacturing industry	246	13.27	195	15.59
Service industry	1127	60.79	807	64.51
Other categories	135	7.28	97	7.75
Annual income (log) ^1^	1854	4.52 (0.29)	1251	4.48 (0.29)
Perceived harmfulness of secondhand smoke (SHS)				
No	268	14.46	164	13.11
Yes	1586	85.54	1087	86.89
Living with child(ren)				
No	1571	84.74	1038	82.97
Yes	283	15.26	213	17.03
Housing type				
Storied commercial building	1166	62.89	853	68.19
Unusual housing type	688	37.11	398	31.81
Number of people sharing one bedroom				
≤2 people in one bedroom	1318	71.09	928	74.18
≥3 people in one bedroom	536	28.91	323	25.82
Availability of facilities				
Availability of facilities	1046	56.42	770	61.55
Lack of facilities	808	43.58	481	38.45
Perceived neighborhood hygiene problem ^1^	1854	2.40 (0.87)	1251	2.41 (0.88)
Perceived neighborhood noise problem ^1^	1854	2.88 (0.99)	1251	2.86 (0.99)
Living in poor hygiene cluster areas				
Yes	191	10.30	114	9.11
No	1663	89.70	1137	90.89
Living in high-level noise areas				
Yes	175	9.44	111	8.87
No	1679	90.56	1140	91.13
Neighborhood greenness				
Mean Normalized Difference Vegetation Index ≤ 0.3	833	44.93	554	44.28
Mean Normalized Difference Vegetation Index > 0.3	1021	55.07	697	55.72
SHS exposure at home				
Never	-	-	682	54.52
1–3 days per week	-	-	109	8.71
4–6 days per week			37	2.96
Every day			423	33.81

^1^ The mean and (SD) are shown for continuous variables.

**Table 2:** Unadjusted odds ratios (ORs) of housing conditions and neighborhood physical environments on SHS exposure at home among non-smoking migrant workers. C.I.: confidence interval. NDVI: Normalized Difference Vegetation Index.

Variables	OR	95% C.I. Low	95% C.I. High
**Housing conditions**			
Unusual housing type	2.09	1.66	2.63
≥3 people in one bedroom	2.13	1.67	2.71
Lack of facilities	1.67	1.34	2.08
**Perceived neighborhood physical environment problems**			
Perceived neighborhood hygiene problem	1.23	1.09	1.40
Perceived neighborhood noise problem	1.17	1.05	1.31
**Neighborhood physical environment based on scan statistics**			
Not living in poor hygiene cluster areas	0.48	0.33	0.69
Not living in high-level noise cluster areas	0.35	0.24	0.51
**Neighborhood greenness based on sensor data**			
Neighborhood mean NDVI value > 0.3	0.78	0.62	0.96

**Table 3:** Partially adjusted odds ratios (ORs) of housing conditions and neighborhood physical environments on SHS exposure at home among non-smoking migrant workers. C.I.: confidence interval. NDVI: Normalized Difference Vegetation Index.

Variables	OR	95% C.I. Low	95% C.I. High
**Housing conditions**			
Unusual housing type	1.77	1.35	2.33
≥3 people in one bedroom	1.77	1.35	2.32
Lack of facilities	1.37	1.06	1.77
**Perceived neighborhood physical environment problems**			
Perceived neighborhood hygiene problem	1.23	1.08	1.41
Perceived neighborhood noise problem	1.17	1.04	1.31
**Neighborhood physical environment based on scan statistics**			
Not living in poor hygiene cluster areas	0.54	0.36	0.81
Not living in high-level noise cluster areas	0.50	0.31	0.80
**Neighborhood greenness based on sensor data**			
Neighborhood mean NDVI value > 0.3	0.89	0.70	1.12

Note: estimated OR for each independent variable comes from the ordered logistic regression model that controlled for all sociodemographic characteristics included in Table 1.

**Table 4:** Fully adjusted odds ratios (ORs) of housing conditions and neighborhood physical environments on SHS exposure at home among non-smoking migrant workers with the neighborhood physical environment scan statistics excluded. C.I.: confidence interval. NDVI: Normalized Difference Vegetation Index.

Variables	OR	95% C.I. Low	95% C.I. High
**Housing conditions**			
Unusual housing type	1.68	1.22	2.31
≥3 people in one bedroom	1.73	1.26	2.38
Lack of facilities	0.95	0.70	1.29
**Perceived neighborhood physical environment problems**			
Perceived neighborhood hygiene problem	1.17	1.02	1.34
Perceived neighborhood noise problem	1.13	1.01	1.27
**Neighborhood greenness based on sensor data**			
Neighborhood mean NDVI value > 0.3	0.92	0.72	1.17

Note: estimated OR for each independent variable comes from the ordered logistic regression model that controlled for all sociodemographic characteristics included in Table 1.

**Table 5:** Fully adjusted odds ratios (ORs) of housing conditions and neighborhood physical environments on SHS exposure at home among non-smoking migrant workers with poor neighborhood physical environments excluded. C.I.: confidence interval. NDVI: Normalized Difference Vegetation Index.

Variables	OR	95% C.I. Low	95% C.I. High
**Housing conditions**			
Unusual housing type	1.64	1.19	2.27
≥3 people in one bedroom	1.72	1.30	2.28
Lack of facilities	0.89	0.65	1.21
**Neighborhood physical environment based on scan statistics**			
Not living in poor hygiene cluster areas	0.62	0.41	0.95
Not living in high-level noise cluster areas	0.58	0.36	0.93
**Neighborhood greenness based on sensor data**			
Neighborhood mean NDVI value > 0.3	0.91	0.71	1.15

Note: estimated OR for each independent variable comes from the ordered logistic regression model that controlled for all sociodemographic characteristics included in Table 1.
